# Microbial Metabolic Pathways for Synergistic Biomethane Augmentation and CO_2_ Sequestration in Coalbed Systems: A Mini-Review

**DOI:** 10.3390/microorganisms14030566

**Published:** 2026-03-02

**Authors:** Yang Li, Longxi Shuai, Qian Zhang

**Affiliations:** 1 Key Laboratory for Prevention of Mine Geological Disasters, Anhui University of Science and Technology, Huainan 232001, China; 2 School of Earth and Environment, Anhui University of Science and Technology, Huainan 232001, China

**Keywords:** coalbed gas bioengineering, hydrogenotrophic methanogens, biomethane, low-negative carbon emission reduction, geological CO_2_ sequestration

## Abstract

Natural gas represents a pivotal transitional clean energy resource, and biogenic coalbed methane (CBM) is ubiquitously distributed in coal reservoirs worldwide. In the context of carbon neutrality targets and the growing demand for large-scale commercial CBM exploitation, innovative technological solutions are urgently required. CBM bioengineering aims to substantially enhance CBM production by stimulating biomethane generation, promoting gas desorption, and improving reservoir permeability, while simultaneously enabling effective CO_2_ sequestration. The potential for biomethane generation is largely governed by the intrinsic physicochemical characteristics of coal, including aromatic structures, maceral composition, and pore–fracture architecture. In addition, hydrogeological conditions—such as geothermal gradients, pH variability, and redox potential—play critical roles in regulating microbial functional gene expression and metabolic enzyme synthesis. Core pretreatment strategies in coalbed gas bioengineering can be broadly classified into approaches that enhance coal bioconversion potential and those that optimize functional microbial consortia. Electric fields and conductive materials can influence microbial community structure by enriching electroactive microorganisms and facilitating interspecies electron transfer. In addition to engineered conductive interventions, reservoir environmental conditions also play an important role in shaping methanogenic community structure. Experimental observations under reservoir-relevant CO_2_ pressure and temperature conditions indicate that deep coalbed environments are associated with shifts in methanogenic community composition, including an increased relative abundance of hydrogenotrophic methanogens. These observations suggest that physicochemical conditions in deep coal seams may favor hydrogen-dependent CO_2_ reduction pathways, thereby supporting hydrogenotrophic methanogenesis and contributing to biomethane generation. The integration of supercritical CO_2_ with microbially acclimated stimulation fluids as an innovative reservoir fracturing strategy offers multiple advantages, including effective reservoir stimulation, permanent carbon sequestration, and sustainable biomethane generation. Future research should focus on modulating coal matrix bioavailability, optimizing microbial consortia, enhancing interspecies metabolic synergies, and advancing carbon fixation bioprocesses to facilitate the large-scale implementation of coalbed gas bioengineering systems. This review synthesizes recent advances in microbially mediated CBM enhancement and CO_2_ sequestration, with a particular focus on field-scale evidence and the key challenges that must be addressed for large-scale implementation.

## 1. Introduction

Fossil fuels remain the dominant global energy source, and China’s coal-dominated energy structure is expected to persist in the foreseeable future. Accordingly, the accelerated development of low-carbon utilization technologies and effective emission reduction strategies for coal resources has become both a critical pathway and an urgent priority for achieving carbon neutrality goals. Although natural gas is widely recognized as an important lower-emission transitional fuel, the development of conventional natural gas resources in China is constrained by geological limitations and uneven resource distribution. As a result, unconventional methane resources, particularly coalbed methane (CBM), have attracted increasing attention as alternative domestic methane sources [1]. CBM represents a coal-associated methane resource that can supplement conventional natural gas supplies and support lower-carbon energy utilization compared with direct coal combustion. China possesses abundant coal resources and substantial CBM potential, providing favorable conditions for further resource development. In addition, the spatial overlap between CBM resources and major coal-producing regions may facilitate cost-effective exploitation through the utilization of existing infrastructure [2]. In recent years, CBM has become an increasingly important supplementary methane resource, and notable progress has been achieved in exploration and production technologies [2]. Studies of deep CBM reservoirs have identified favorable geological characteristics, including high gas content and suitable pressure–storage conditions, indicating significant development potential. However, the exploitation of deep CBM reservoirs remains technically challenging due to complex geological conditions and limitations in effective reservoir stimulation. These challenges have stimulated growing interest in alternative enhancement strategies, including microbial processes that contribute to methane generation and reservoir transformation. Importantly, under elevated temperature–pressure conditions, the progressive increase in free and dissolved gas concentrations with depth suggests the potential formation of discrete water-soluble gas accumulations. This phenomenon not only represents an emerging exploration target for unconventional energy systems, but also implies the presence of unique subsurface environments where gas generation, storage, and transformation processes may differ fundamentally from those in shallow reservoirs [3].

To clarify the role of microorganisms in CBM systems, biogenic and thermogenic CBM are commonly distinguished based on their origin. Thermogenic CBM accumulations are generated through thermal cracking of organic matter under deep burial and high-temperature conditions, where microbial communities are generally less abundant and exhibit lower metabolic activity; consequently, microbial stimulation strategies are considered less applicable under such conditions. In contrast, biogenic CBM is generated and sustained by active microbial consortia under relatively shallow burial and low thermal maturity conditions. In these environments, hydrolytic bacteria and methanogenic archaea are widely distributed, and both their abundance and community diversity are often associated with methane occurrence and production potential. Importantly, microbial activity in biogenic CBM systems can be influenced and potentially enhanced through targeted microbial management strategies to support sustained methane production [4,5]. Strategic activation of microbial metabolic processes in coal reservoirs therefore represents a promising approach for substantially improving CBM recovery, thereby unlocking critical development potential for this unconventional energy resource. Recent research in CBM bioengineering has increasingly examined microbial acclimation strategies and environmental controlling factors that influence microbial activity and methane production [6]. Microbial activity has also been reported to influence the pore–fracture structure of coal matrices, which may improve conditions for methane migration and recovery. In addition, the integration of Carbon Capture, Utilization and Storage (CCUS) with CBM bioengineering has attracted growing attention as a potential approach for coupling biomethane production with geological carbon sequestration [5]. Studies have shown that carbon dioxide injection into coal reservoirs may influence microbially mediated metabolic pathways, potentially enhancing hydrogenotrophic methanogenesis while contributing to CO_2_ storage within coal formations. This coupled process highlights the potential for integrating microbial methane production enhancement with carbon sequestration strategies in CBM systems [7,8].

## 2. Synergistic Microbial Consortia Drive Carbon Substrate Conversion and Methanogenic Metabolism

**Figure 1:**
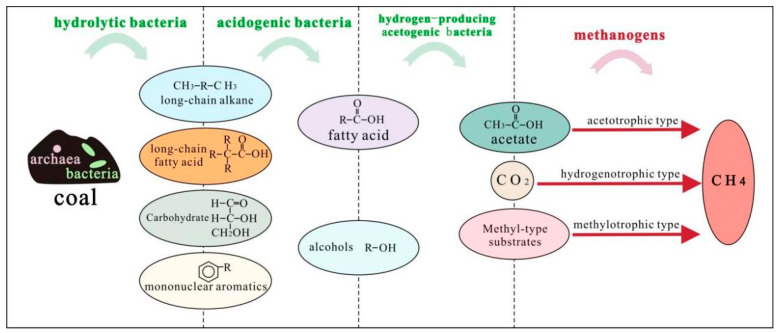
Schematic diagram of methane production from coal co-degradation by microbial consortia.

As shown in Figure 1, the bioconversion of organic substrates to methane in coal reservoirs proceeds through a multi-stage biochemical process driven by dynamic microbial consortia. This complex metabolic cascade requires the coordinated activities of diverse functional microbial groups, including hydrolytic bacteria, acidogenic bacteria, hydrogen-producing acetogenic bacteria, and methanogenic archaea, which sequentially mediate substrate degradation through tightly coupled metabolic interactions. In CBM reservoirs, these metabolic pathways operate as an interdependent cascade rather than as isolated stages, with the efficiency of each step influenced by microbial community structure and the physicochemical properties of the coal seam. The hydrolysis of complex coal-derived organic matter by hydrolytic bacteria plays a critical role in determining substrate availability for downstream acidogenic and acetogenic processes. However, the overall rate of the cascade is strongly influenced by the syntrophic oxidation of intermediate compounds to acetate and hydrogen, which is mediated by hydrogen-producing acetogens and depends on the efficient consumption of hydrogen by methanogenic archaea to maintain thermodynamic feasibility. Consequently, methanogenic activity is a key determinant of methane yield and plays a central role in regulating metabolic flux within the microbial network. This syntrophic interaction is fundamental to biogenic CBM formation and represents an important target for microbial enhancement approaches.

The catalytic activity of hydrolytic bacteria is widely recognized as an important factor influencing anaerobic digestion processes and contributes to the efficiency of microbially mediated coal degradation. Among the successive stages of hydrolysis, including extracellular enzyme secretion, enzyme adsorption, macromolecular cleavage, and the release of soluble products, the initial secretion of extracellular enzymes is commonly considered one of the slower steps and is often regarded as a key rate-limiting factor influencing downstream methane production. Through the secretion of diverse extracellular enzymes, hydrolytic microbial consortia facilitate the depolymerization of complex aromatic macromolecules containing bridge bonds and aliphatic side chains [9,10]. Extracellular enzymes mediate the hydrolysis of carbohydrates into simple sugars, while lipases catalyze the conversion of lipids into glycerol and fatty acids. Proteolytic enzymes promote the breakdown of proteins into amino acids, which may undergo further transformation into long-chain alkanes and monocyclic aromatic hydrocarbons. In such systems, these compounds are generated concurrently as hydrolysis products rather than occurring exclusively through a strictly sequential transformation pathway. The ecological stability and functional performance of hydrolytic communities therefore depend on dynamic interactions among functional groups. Competitive utilization of limited substrates can influence carbon flux distribution and the availability of key intermediates, such as acetate, hydrogen, and formate, thereby affecting downstream methanogenic activity [11]. In contrast, cooperative cross-feeding of formate and hydrogen between syntrophic acetogens and hydrogenotrophic methanogens can enhance carbon dioxide reduction and may contribute to increased methane production by maintaining low hydrogen partial pressure and thermodynamically favorable conditions for interspecies electron transfer [12]. Such interspecies metabolic interference highlights the necessity for careful optimization in biotechnological applications, including targeted functional strain enrichment, diversification of microbial resources, and the integration of adaptive laboratory evolution with field-scale process intensification strategies [13]. In the context of CBM development, hydrolysis is widely recognized as the principal rate-limiting pathway in biogenic conversion, particularly in high-rank coal seams characterized by densely cross-linked aromatic macromolecular structures. The activity of extracellular hydrolytic enzymes is highly sensitive to coal seam physicochemical conditions, especially pH and temperature, such that even moderate deviations from optimal ranges can lead to pronounced declines in substrate degradation efficiency. Consequently, enhancing hydrolytic bacterial activity—through in situ nutrient amendment or the introduction of enzyme-enriched microbial consortia—has become a critical and extensively investigated strategy for improving biogenic CBM production.

Acidogenic bacteria further metabolize the byproducts generated during hydrolysis and primary fermentation into a range of low-molecular-weight compounds, including propionic acid, butyric acid, and various alcohols. This acidogenic phase is characterized by a progressive decrease in system pH and an increase in the concentration of reducing substances, reflecting intensified microbial redox activity [14]. During acidogenic fermentation, organic substrates with relatively low bond dissociation energy are preferentially utilized, resulting in enhanced metabolic turnover. This stage primarily involves the degradation of straight-chain and branched-chain hydrocarbons. Straight-chain alkanes are initially activated through carboxylation reactions, leading to the formation of fatty acids and pyruvate intermediates [15]. In parallel, carbohydrates are metabolized via glycolytic pathways to generate pyruvate. Pyruvate serves as a central metabolic node during this phase and undergoes decarboxylation catalyzed by pyruvate–ferredoxin oxidoreductase, yielding reduced fermentation products such as propionic acid and butyric acid. Alternatively, pyruvate can be redirected into a series of enzymatic reactions producing neutral metabolites, including 2,3-butanediol. As acidogenic fermentation proceeds, concentrations of alkanes and carbohydrates decline markedly, while pyruvate exhibits a transient accumulation followed by gradual depletion, reflecting its rapid turnover within interconnected metabolic pathways [16]. Short-chain fatty acids and alcohols produced during acidogenesis constitute key intermediates supporting downstream methanogenesis, contributing to the acetate pool and molecular hydrogen consumed by methanogenic archaea. Accordingly, the acidogenic phase plays a central role in linking primary substrate degradation to methane formation. However, excessive accumulation of volatile fatty acids (VFAs) can lead to acidification of coal seam water, which may suppress hydrolytic enzyme activity and inhibit methanogen growth. The balance between VFA production and consumption therefore reflects microbial ecosystem stability in CBM reservoirs. Maintaining this balance is important for sustaining microbial activity and supporting stable methane production in CBM systems.

Hydrogen-producing acetogenic bacteria catalyze the further conversion of volatile fatty acids into a suite of low-molecular-weight metabolites, including formate, acetate, methanol, methanethiol, hydrogen, and CO_2_. Within anaerobic ecosystems, these syntrophic microorganisms occupy a pivotal metabolic position, functioning as intermediaries that link acidogenic fermentative bacteria with methanogenic archaea. Notably, certain acidogenic taxa exhibit metabolic versatility, simultaneously operating hydrogenogenic and acetogenic pathways that facilitate the sustained transformation of hydrolysis–fermentation products into acetate, hydrogen, and CO_2_ [17]. During the hydrogen-producing acetogenic stage, microbial communities actively mediate the structural modification of aromatic compounds through oxidative cleavage of alkyl side chains. This transformation introduces oxygen-containing functional groups, such as hydroxyl, carbonyl, and ester moieties, while yielding aliphatic chain derivatives. In parallel, microbial dissimilation pathways convert reduced intermediates, including 2,3-butanediol, into 2,3-butanedione and related compounds, ultimately channeling carbon flux toward acetate formation as the dominant metabolic end product [18]. By sustaining hydrogen and formate fluxes and alleviating electron sink limitations associated with CO_2_ reduction, syntrophic acetogenesis contributes electron donors for hydrogenotrophic methanogenesis and supports downstream methanogenic activity. Hydrogen-producing acetogenic bacteria represent an important metabolic link between acidogenesis and methanogenesis in CBM reservoirs and help alleviate thermodynamic constraints associated with interspecies electron transfer [11,12]. In deep CBM reservoirs characterized by elevated hydrostatic pressure and limited gas diffusion, direct interspecies electron transfer (DIET) has been proposed as an alternative mechanism for electron exchange between syntrophic partners, in addition to hydrogen-mediated transfer. This DIET-based interaction may improve energetic efficiency and stability of syntrophic metabolism and may contribute to microbial interactions in deep biogenic CBM systems.

Methanogenic archaea employ a suite of specialized coenzymes to catalyze the conversion of CO_2_, acetate, and methylated compounds into methane through distinct but interconnected metabolic pathways. Based on substrate utilization patterns and enzymatic systems, methanogens are phylogenetically and functionally classified into hydrogenotrophic (CO_2_-reducing), acetotrophic, and methylotrophic groups. Hydrogenotrophic methanogenesis proceeds via the stepwise reduction of CO_2_ to methane, using hydrogen or formate as electron donors and relying on tightly coupled electron bifurcation mechanisms to conserve energy. In contrast, acetotrophic methanogenesis involves the cleavage of acetate into methyl and carboxyl moieties, with oxidative decarboxylation of the carboxyl group supplying reducing equivalents for the subsequent reduction of the methyl group. Methylotrophic methanogens utilize simple methylated substrates through dedicated methyltransferase systems, requiring either external electron donors or internal redox coupling among methyl groups to achieve methane formation [18,19]. In engineered biostimulation of low-rank CBM reservoirs, hydrogenotrophic methanogenesis, driven by hydrogen-dependent CO_2_ reduction, is often observed as a major methane-producing pathway, particularly under conditions where hydrogen or formate availability is elevated. As a result, this pathway is considered an important target for CBM biostimulation strategies, especially in systems where hydrogen availability supports microbial CO_2_ reduction. Methanogens are obligate anaerobes with stringent physiological constraints, including low biomass yield, slow growth rates, and narrow substrate spectra. In practical anaerobic systems, methanogenic performance is often limited by insufficient substrate fluxes and electron donor availability, as well as by the difficulty of maintaining favorable redox conditions for sustained activity [9,10]. Ecological surveys further reveal pronounced spatial and temporal heterogeneity in methanogenic community composition and abundance, driven by substrate distribution, syntrophic interactions, and thermodynamic constraints on key metabolic intermediates. Within this context, bacterial community structure and the availability of metabolic intermediates play decisive roles in shaping methanogenic dynamics and population succession [20,21]. The three principal methanogenic pathways exhibit distinct adaptive patterns across CBM reservoirs. Hydrogenotrophic methanogenesis is frequently dominant in deeper, higher-temperature coal seams, whereas acetotrophic methanogenesis is more commonly associated with shallow, low-rank coal seams. Methylotrophic methanogenesis generally contributes a smaller proportion of methane production and is typically restricted to specific geochemical or contamination-influenced environments. This pathway differentiation is largely influenced by coal rank, substrate availability, and prevailing geochemical conditions, providing an important theoretical basis for the rational design of targeted CBM biostimulation strategies. By identifying the predominant methanogenic pathway within a given coal reservoir, nutrient amendment schemes and microbial management approaches may be adjusted to selectively support key methanogenic consortia, thereby potentially enhancing in situ biogenic methane generation.

## 3. Key Factors Affecting Microbial Metabolism and Coal Biodegradation Processes

Coal exhibits an exceptionally complex macromolecular matrix composed of diverse organic compounds that are inherently resistant to microbial utilization. Although low-molecular-weight organic compounds susceptible to biodegradation are present, they are often physically entrapped within this dense matrix, thereby limiting microbial accessibility and metabolic conversion [22,23]. While coals of all ranks are subject to microbial degradation, low-rank coals generally contain a higher abundance of labile aliphatic side chains, whereas high-rank coals are dominated by condensed aromatic structures that are more recalcitrant to biodegradation [24,25,26]. Consequently, disruption of the coal matrix and cleavage of aromatic carbon–carbon bonds can markedly enhance coal bioavailability and microbial conversion efficiency [27]. Biomethane production potential is further influenced by coal maceral composition. Vitrinite is characterized by relatively low degrees of aromatization and a higher proportion of reactive carbon structures, whereas inertinite consists predominantly of highly aromatized and structurally condensed carbon frameworks. As a result, vitrinite-rich coals are often associated with greater biomethane generation potential compared with inertinite-rich counterparts, indicating that aromatization degree and aliphatic hydrocarbon content are important factors influencing methane yield [28,29,30]. Under comparable nutrient amendment conditions, low-rank coals generally show higher biomethane production potential than high-rank coals. This difference is commonly associated with their lower aromaticity, higher volatile matter content, and more developed mesoporous structures, which may improve microbial accessibility and substrate availability. In addition to chemical composition, the physical structure of coal—including porosity, permeability, particle size, and specific surface area—plays a critical role in regulating microbial colonization and metabolic activity [31,32]. In intact hard coal reservoirs where primary pore networks are poorly connected, hydraulic fracturing can create secondary fluid migration pathways and expand habitable space for microorganisms, which preferentially colonize coal macropores and fracture systems [33,34,35].

Microbial reactions are fundamentally governed by enzyme-catalyzed processes, the efficiency of which is tightly regulated by environmental conditions. Key physicochemical factors—including temperature, pH, and redox potential—exert strong control over enzyme synthesis, catalytic activity, and the expression of functional genes within microbial communities [36,37,38]. In coal reservoirs, temperature generally increases with burial depth, and deeper seams often provide thermal conditions conducive to sustained microbial metabolism. Under elevated temperature regimes, methanogens with metabolic flexibility tend to progressively favor hydrogenotrophic pathways over alternative methanogenic routes [39,40]. pH influences microbial activity not only by regulating nutrient transport through alterations in cell membrane charge but also by directly modulating enzyme conformation and catalytic efficiency [41]. Redox potential serves as an integrated indicator of the oxidative state of microbial habitats, with oxygen and other oxidizing agents elevating redox levels. Microorganisms exhibit distinct redox tolerances, and methanogens in particular preferentially thrive under strongly reducing conditions, whereas elevated redox potentials can cause irreversible oxidative damage to key enzymatic systems [42]. Trace elements constitute another critical environmental constraint, acting as essential nutrients for microbial growth and as structural or catalytic components of enzymes involved in anaerobic metabolism. Multivalent iron, for example, functions both as an electron carrier and as a cofactor in multiple enzymatic complexes central to anaerobic fermentation and methanogenesis [43,44]. These environmental parameters interact in a closely coupled and potentially synergistic manner. Moderate temperatures help maintain enzymatic activity, near-neutral pH conditions are associated with improved membrane transport and cofactor stability, low redox potential may reduce oxidative stress on methanogenic enzymes, and adequate trace element availability supports the supply of electron carriers required for processes such as acetate cleavage and CO_2_ reduction. Accordingly, field-scale applications may benefit from considering these factors as components of an integrated hydrogeochemical system, as perturbations beyond the tolerance range of individual parameters may influence system stability and potentially affect the effectiveness of microbial enhancement strategies for CBM production.

**Figure 2:**
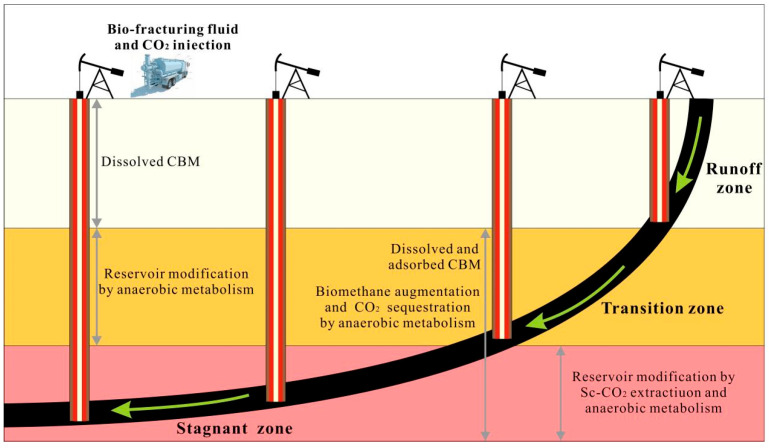
Technical model of biomethane augmentation and CO_2_ sequestration in coalbed systems.

Biogenic CBM formed over geological time has experienced multiple evolutionary stages, including coalification, tectonic uplift, and hydrodynamic modification. During these processes, surface water and meteoric precipitation transport nutrients and microorganisms into coal seams, where hydrolytic fermentative bacteria and methanogenic archaea can colonize and degrade coal under suitable physicochemical conditions, leading to methane generation and preservation [45,46,47]. Subsequent tectonic uplift or episodic aquifer recharge may reactivate dormant microbial communities, indicating that biogenic CBM reservoirs often represent the cumulative outcome of multiphase biomethane generation and preservation within coal-bearing strata. Groundwater flow plays a central role in this process by supplying essential substrates to microbial communities, while groundwater characteristics—including temperature, pH, redox conditions, and trace element availability—directly regulate microbial metabolism and reproduction [48,49]. As illustrated in Figure 2, groundwater systems can be vertically subdivided into runoff, transitional, and stagnant zones based on hydrodynamic conditions. The runoff zone commonly occurs in steeply dipping, near-surface settings and is characterized by rapid recharge and relatively oxidizing conditions, under which microbial communities are unable to fully exploit available nutrients or establish the strongly anaerobic environments required for sustained methanogenesis. In contrast, the stagnant zone typically develops in gently dipping areas distal from recharge sources and is marked by slow groundwater movement and relatively reducing conditions that are favorable for microbial metabolism. However, limited hydrodynamic exchange in this zone can restrict nutrient replenishment or lead to substrate depletion over time [50,51]. Consequently, the interval spanning the stagnant-to-transitional zones is widely regarded as one of the most favorable settings for microbial CBM enhancement. In these zones, slow yet persistent groundwater flow can facilitate sustained nutrient transport, reducing conditions support methanogenic activity, and moderate temperatures help maintain enzymatic stability and metabolic efficiency. These environmental factors function as components of an integrated hydrodynamic–biogeochemical system, whose effectiveness depends strongly on mutual coupling rather than solely on isolated optimization. Favorable microbial habitats are therefore often associated with specific reservoir depths and hydrodynamic regimes that balance anaerobic conditions with continuous nutrient delivery. Such environmental characteristics may help explain, at least in part, the pronounced enrichment of biogenic CBM observed in coal reservoirs such as the Powder River Basin [52,53]. When microbial metabolic activity remains synchronized with nutrient replenishment cycles, efficient methanogenesis and CO_2_ reduction can be sustained. Following periods of microbial quiescence, surface drainage-induced depressurization, in combination with bio-fracturing fluids or CO_2_ injection, may facilitate reactivation and directional regulation of these biogenic processes [46,47,48].

## 4. Pretreatment Strategies for Coalbed Gas Bioengineering

The availability of bioaccessible organic substrates and the metabolic performance of carbon-degrading microbial consortia are widely regarded as important factors influencing the effectiveness of CBM bioengineering, particularly during the pretreatment stage. Accordingly, current research in this field has focused on strategies targeting both the coal substrate and the associated microbial communities. One line of investigation aims to enhance the bioconversion potential of coal through mild oxidative or physicochemical pretreatment approaches, which have been examined primarily under controlled experimental conditions. In parallel, increasing attention has been directed toward improving the structural stability and functional performance of microbial consortia, including the development of synthetic microbial communities and metabolic regulation strategies. Together, these approaches define the principal research directions currently being explored to improve CBM bioengineering performance.

### 4.1. Enhancement of Coal Bioconversion Potential

Enhancement of coal bioavailability primarily relies on physicochemical pretreatment strategies designed to increase microbial access to organic constituents within the coal matrix. Physical approaches include hydraulic fracturing, microwave irradiation, and supercritical CO_2_ (Sc-CO_2_) extraction [54,55]. Hydraulic fracturing improves reservoir permeability by modifying pore–fracture networks, thereby enlarging the reactive surface area available for microbial colonization and substrate exchange. Microwave pretreatment exhibits a dual effect by promoting mineral removal and inducing microfracture development; however, its large-scale field application remains constrained by technological and operational challenges. Sc-CO_2_ pretreatment operates through molecular-scale interactions, weakening non-covalent forces within coal macromolecular networks and facilitating the release of low-molecular-weight organic compounds via attenuation of intermolecular interactions [56,57]. In contrast, chemical pretreatment strategies aim to directly depolymerize macromolecular organic matter into smaller, more bioavailable substrates. Following chemical treatment, coal structures become more accessible to hydrolytic and fermentative microorganisms, thereby enhancing downstream biodegradation processes [58,59,60]. A range of chemical agents, including acids, alkalis, oxidants, surfactants, and chelating agents, have been reported, primarily under laboratory and pilot-scale conditions, to promote the liberation of low-molecular-weight organics, disrupt aromatic structures, and increase the abundance of oxygen-containing functional groups. Collectively, these transformations can enhance the availability of dissolved organic carbon, volatile fatty acid precursors, and enzymatically accessible substrates [61]. Oxidants and reagents such as H_2_O_2_, O_3_, KMnO_4_, HNO_3_, NaOH, and CH_3_COOOH have demonstrated varying degrees of effectiveness under specific experimental conditions. However, acid- and alkali-based pretreatments may alter groundwater chemistry, potentially increasing salinity, mobilizing heavy metals, and generating residual oxidants that inhibit microbial activity, depending on reservoir geochemical characteristics. These environmental risks necessitate careful evaluation prior to field-scale deployment, as enhanced coal bioavailability must be balanced against operational feasibility and potential remediation requirements [62,63,64,65]. Among the available oxidants, H_2_O_2_ has been explored as a candidate for in situ applications due to its strong oxidative capacity and rapid decomposition. Nevertheless, its application can transiently suppress microbial activity and introduce oxygen into the system, which may disrupt native anaerobic conditions. In such cases, additional measures, including microbial reinjection or stimulation, may be required to facilitate the recovery of reducing conditions conducive to methanogenesis [66,67,68]. The microbial degradation of coal serves dual functions in CBM development. It not only enhances gas resource potential and production capacity but also fundamentally modifies reservoir characteristics through structural reorganization of coal’s pore-fracture architecture. This bioconversion process facilitates optimized gas desorption, matrix expansion, and permeability enhancement through three key mechanisms [69,70]. In terms of pore structure evolution, microbial activity induces scale transformation, converting nanometer-scale pores into micrometer-scale dimensions. Concurrently, organic compound deposition partially occludes nanoscale voids, further promoting micron-scale porosity dominance. In terms of mechanical modification, microbial weakening of pore wall integrity creates interconnections between previously isolated nanopores, resulting in increased total pore volume, frequency distribution shifts toward larger diameters, and enhanced void connectivity. In terms of surface modification, treatment reduces pore surface roughness and specific surface area, effectively diminishing the reservoir’s methane adsorption capacity while improving migration pathways [71,72]. These synergistic modifications establish favorable reservoir conditions for methane desorption from coal matrices and subsequent gas accumulation in fracture networks [73,74].

### 4.2. Optimization of Functional Microbial Consortia

Microbial consortia indigenous to coal reservoirs are often insufficient to support methane production at scales required for commercial optimization. Enhancement of functional microbial consortia therefore relies on the targeted enrichment of high-efficiency strains, which is most commonly achieved through optimization of culture media formulations [75,76]. Post-domestication analyses indicate that such enrichment is frequently accompanied by increased expression of functional genes related to membrane transport and central metabolism, leading to restructuring of key microbial metabolic networks [77,78]. Current research on microbial cultivation primarily focuses on the domestication of individual strains or mixed consortia, optimization of nutrient composition and concentration in growth media, and evaluation of mineral cofactors and trace elements that regulate enzymatic performance. Coordinated activation of microbial enzymatic functions across different metabolic stages has been associated with shorter lag phases of methanogenesis and improved efficiency of terminal metabolic processes under controlled experimental conditions [79,80]. In parallel, co-metabolism strategies involving coal and supplementary biomass, such as straw, have been reported, under specific experimental settings, to promote coal depolymerization, alter microbial community structures, and influence dominant methanogenic pathways. Although biomass-derived low-molecular-weight organics are readily assimilated by microbes and may facilitate coal biotransformation, the underlying molecular mechanisms remain insufficiently resolved [81,82,83]. Reported field-scale microbial enhancement trials have involved reservoir inoculation with enriched microbial consortia or the injection of nutrient-amended culture fluids [84,85,86,87]. However, practical implementation remains constrained by several factors, including declines in the viability of injected microorganisms, competitive interactions with indigenous microbial communities, and the difficulty of sustaining metabolic activity under dynamically evolving reservoir conditions. These limitations underscore the importance of developing more robust and adaptable strategies. In this context, the development of region-specific, cost-effective microbial formulations and cultivation media tailored to local geological settings and engineering objectives is increasingly regarded as a promising direction for future CBM biotechnological applications [87,88,89].

**Figure 3:**
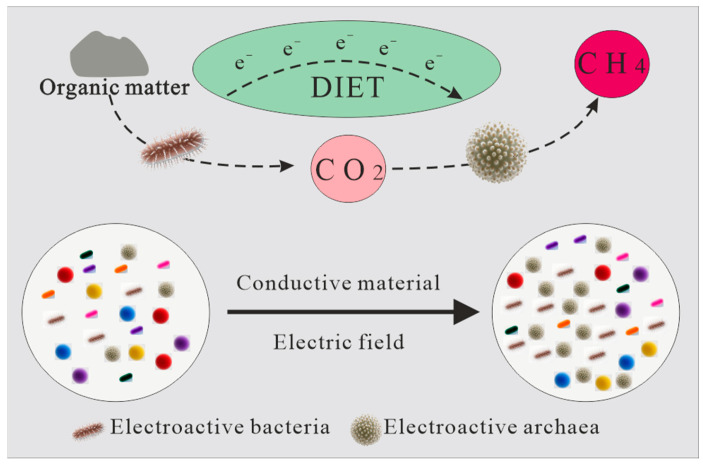
Schematic diagram showing biomethane augmentation and CO_2_ sequestration through electromethanogenesis.

Electron supply and transfer constitute central regulators of synergistic microbial metabolism and bioenzyme gene expression within CBM systems. Key constraints, including limited electron donor availability, inefficient electron flux, and the scarcity of electroactive functional microorganisms, restrict the degradation of coal-derived organic matter and disrupt the continuity of methanogenic metabolism [90,91]. Electroactive microorganisms facilitate extracellular electron transport through outer-membrane cytochrome networks or conductive pili, enabling interspecies electron transfer (IET) that enhances collective electron utilization efficiency. Among these mechanisms, direct interspecies DIET refers specifically to carrier-free electron exchange between microbial partners or between microorganisms and conductive materials or electrode interfaces [92,93]. As illustrated in Figure 3, strategies to enhance DIET primarily include the application of external electric fields to activate thermodynamically constrained redox reactions, as well as the incorporation of conductive additives that stimulate extracellular electron transfer pathways, promote functional gene expression, and enrich electroactive microbial populations [94,95]. Representative electroactive taxa include bacterial families such as *Bacillaceae*, *Bacteroidaceae*, and *Clostridiaceae*, as well as genera *Geobacter* and *Pseudomonas*, in addition to methanogenic archaea including *Methanosarcina* and *Methanosaeta*. Coal organic matter, dominated by lignocellulosic Type III kerogen of terrestrial origin, can be efficiently metabolized by these electroactive bacteria through coupled aromatic compound and carbohydrate degradation pathways. Although native microbial pili generally exhibit limited conductivity, engineered conductive materials can establish more efficient DIET networks with methanogens, thereby strengthening syntrophic interactions. Carbon-based conductive materials, including graphite, graphene, and biochar, have demonstrated substantial potential for enhancing DIET under anaerobic conditions [96,97]. Graphite exhibits favorable mechanical stability and density characteristics that are broadly compatible with subsurface deployment requirements and share certain functional similarities with conventional hydraulic fracturing proppants such as quartz sand. Its physicochemical stability under elevated pressure, shear, and dynamic flow conditions, together with favorable suspension characteristics, may facilitate subsurface transport and distribution within fracture networks. Experimental studies have reported that electroactive bacterial taxa, including Pseudomonas species, may participate in DIET interactions with methanogens such as Methanosaeta in the presence of conductive materials such as graphite, contributing to enhanced methane production and CO_2_ reduction efficiency under controlled conditions [97,98]. Despite these advantages, field-scale application remains constrained by several unresolved challenges, including uncertainties in long-distance subsurface transport, long-term material stability under cyclic mechanical stress and variable salinity conditions, and the development of cost-effective delivery strategies. The integration of Sc-CO_2_ extraction with graphite-assisted DIET systems has been proposed as a potentially promising approach for concurrent enhancement of methane production and carbon management. However, the mechanisms governing subsurface material migration and long-term functional persistence require further systematic investigation.

## 5. Advances in Microbial-Mediated CO_2_ Sequestration in CBM Reservoirs

The feasibility of CO_2_-enhanced CO_2_-ECBM recovery is fundamentally controlled by the preferential adsorption of CO_2_ relative to methane on coal matrices, in conjunction with reservoir pressure evolution during injection and production. Effective displacement generally requires CO_2_ injection volumes that substantially exceed the in situ methane inventory. Following injection, CO_2_ is sequestered through dual mechanisms, including dissolution into coal seam brines and adsorption onto micropore surfaces within the coal matrix [99]. Existing CBM production infrastructure, such as wellbores and pipeline networks, provides a practical foundation for coupling CO_2_ storage with enhanced methane recovery operations [100]. Within deep coal-bearing systems, chemolithoautotrophic microbial consortia further mediate CO_2_ transformation through hydrogenotrophic methanogenesis, yielding biomethane, as well as through carbonate mineralization processes that contribute to long-term carbon stabilization. Microbial CO_2_ biomethanation proceeds predominantly via the hydrogenotrophic pathway catalyzed by specialized archaeal communities, with representative genera including *Methanobacterium*, *Methanothermus*, *Methanococcus*, and *Methanosarcina*. This biochemical process requires the coordinated availability of dissolved inorganic carbon, active hydrogenotrophic methanogens, and suitable external electron donors [101,102]. Most coal reservoirs are characterized by strongly reducing conditions, under which methanogenic archaea preferentially assimilate bicarbonate as their primary carbon source. Injected CO_2_ partitions between adsorbed phases on coal surfaces and dissolved phases in formation water, thereby establishing a sustained carbon reservoir that supports microbial metabolism. Observations from biogenic CBM systems worldwide indicate that hydrogenotrophic methanogenesis constitutes the dominant pathway for biomethane generation, with CO_2_ reduction accounting for the majority of documented accumulations [103]. In deep biosphere settings, elevated hydrostatic pressures and moderate thermal regimes further favor hydrogenotrophic methanogen proliferation. Pretreatment approaches that enhance coal bioavailability and optimize functional microbial consortia have the potential to markedly intensify carbon conversion efficiency, thereby strengthening the coupling between CO_2_ sequestration and biomethane generation. Together, these processes position CO_2_-ECBM as a promising pathway toward integrated energy recovery and carbon management, with the potential to contribute to low- or net-negative carbon emission strategies under appropriate geological and operational conditions [104,105].

At depths exceeding 800 m, the prevailing temperature and pressure conditions maintain CO_2_ in a supercritical state. Under these conditions, Sc-CO_2_ exhibits substantially enhanced diffusivity relative to liquid CO_2_, allowing more effective penetration into coal matrices and facilitating the extraction of organic constituents through intensified molecular-scale interactions [106,107]. Recent studies indicate that, despite their metabolic versatility, methanogens preferentially rely on the hydrogenotrophic pathway for methane production under Sc-CO_2_ exposure. Although Sc-CO_2_ extraction increases the availability of carbon substrates for microbial metabolism, it concurrently imposes pronounced physiological stress on microbial cells [108]. Exposure to Sc-CO_2_ can compromise membrane integrity, induce cytoplasmic acidification, and disrupt osmotic balance. Elevated CO_2_ partial pressures further promote intracellular accumulation of reactive nitrogen species, which interferes with essential cellular processes, including protein synthesis, gene regulation, and electron transport. Importantly, these inhibitory effects are not strictly permanent; microbial functionality can be progressively restored during prolonged exposure through adaptive metabolic recalibration and physiological adjustment [108,109]. Microbial consortia may develop tolerance to Sc-CO_2_ conditions through biofilm formation mediated by surface adhesion mechanisms. The resulting extracellular polymeric matrix enhances cellular resistance to physicochemical stress while simultaneously strengthening CO_2_–rock interfacial interactions. This process may contribute to improved formation affinity and reduced CO_2_ mobility, thereby mitigating the risk of reservoir-scale leakage under supercritical injection conditions [110].

Sc-CO_2_ exhibits exceptional molecular penetration capacity, enabling effective modification of micropore structures within coal reservoirs. Post-extraction analyses indicate pronounced structural reorganization across hierarchical pore–fracture networks in coal matrices. The Sc-CO_2_ extraction process reduces the abundance of oxygen-containing functional groups on coal surfaces, which represent key adsorption sites for methane molecules [99]. Concurrently, Sc-CO_2_ treatment lowers pore-network tortuosity and structural complexity, thereby decreasing diffusive resistance to gas transport within the coal matrix.

Pore volumes across multiple spatial scales increase to differing extents following Sc-CO_2_ exposure, with macropores at the micrometer scale exhibiting particularly pronounced development. These enlarged pore domains serve as primary conduits for advective fluid flow. In addition, volumetric expansion associated with CO_2_ phase transitions generates mechanical stress within the coal matrix, which may initiate microfracturing and promote a shift in fluid transport behavior from diffusion-dominated to seepage-controlled regimes. The enhanced solvation capacity of Sc-CO_2_ further facilitates mineral dissolution, contributing to the formation of more interconnected pore networks [106,107]. Following physicochemical extraction, residual coal substrates remain available for microbial utilization. Continued microbial metabolism progressively enhances pore connectivity while further reducing structural complexity and tortuosity. Together, these coupled physicochemical and biological processes improve reservoir permeability, weaken residual methane adsorption capacity, and promote methane desorption from the coal matrix. As illustrated in Figure 2, the mechanistic coupling of Sc-CO_2_ extraction with microbial metabolism produces synergistic enhancement of desorption efficiency, pore-network evolution, and permeability development, highlighting the considerable potential of this integrated strategy for optimized coalbed methane recovery [102,103,104,105,106,107,108,109,110].

Integrated analysis of Sc-CO_2_ temperature–pressure phase behavior and the thermal tolerance of methanogenic microorganisms indicates that mid-deep coal seams satisfying both supercritical conditions and microbial metabolic requirements provide a favorable window for synergistic bio-engineering applications. Under such conditions, Sc-CO_2_-mediated physicochemical extraction can be effectively coupled with microbial conversion processes, enabling simultaneous reservoir modification, enhanced methane recovery, and carbon management within a unified operational framework [105]. Field implementation typically involves staged reservoir stimulation, microbial introduction, and subsequent monitoring of gas composition and isotopic characteristics to evaluate system performance and microbial activity [108,109,110,111]. Among the diagnostic parameters, the carbon isotopic signature of methane (δ^13^C–CH_4_), carbon isotope fractionation between CO_2_ and CH_4_ (αC), and CO_2_/CH_4_ ratios are widely applied to evaluate dominant methanogenic pathways and to assess the persistence of hydrogenotrophic activity [112,113]. Sustained lighter δ^13^C–CH_4_ values together with stable or increasing isotopic fractionation factors provide evidence for continued CO_2_ reduction, whereas long-term trends in CO_2_/CH_4_ ratios and methane production rates serve as indicators of process stability and effective carbon conversion within the reservoir [114,115]. In addition to Sc-CO_2_-driven direct interspecies electron transfer (Sc-CO_2_–DIET), several alternative biogas upgrading strategies have been explored, including in situ hydrogen injection for biological CO_2_ methanation, anaerobic membrane-based CH_4_ enrichment, and microbial electrosynthesis using bioelectrochemical systems. These approaches are still under active investigation and development and may offer complementary pathways for enhancing methane production and CO_2_ utilization. Compared with single-mechanism approaches, integrated strategies combining Sc-CO_2_ stimulation with microbial processes provide the potential to simultaneously influence reservoir properties, microbial activity, and carbon transformation pathways.

Building upon the mechanistic framework outlined above, recent studies have further refined the understanding of CO_2_-driven reservoir modification and microbial carbon conversion under deep coal seam conditions. High-pressure microfluidic visualization experiments and in situ spectroscopic analyses indicate that Sc-CO_2_ injection can induce dynamic wettability shifts and mineral–organic interfacial restructuring, processes that are likely to influence long-term CO_2_ retention and methane displacement behavior [116,117]. Parallel isotopic and high-pressure incubation studies conducted under simulated deep-biosphere conditions suggest that hydrogenotrophic methanogenesis may remain an important metabolic pathway during elevated CO_2_ exposure, particularly under diffusion-limited carbon availability [118]. Laboratory-scale coupling experiments further demonstrate that Sc-CO_2_ pretreatment can enhance coal surface accessibility and pore connectivity, thereby facilitating gas transport and methane desorption dynamics [116,119]. In addition, indigenous microbial activity has been observed to modify pore structures within coal matrices, potentially influencing methane migration behavior [120]. Collectively, these findings suggest the technical feasibility of integrating Sc-CO_2_ stimulation with microbial conversion processes in mid-deep coal reservoirs, while providing experimentally constrained parameters for future field-scale optimization [121]. Nevertheless, several critical challenges remain for large-scale implementation. Reservoir heterogeneity may result in uneven CO_2_ distribution and variable permeability enhancement, potentially affecting methane recovery efficiency and storage performance [121]. Sustained hydrogen availability for hydrogenotrophic methanogenesis may represent an operational constraint, particularly under high-pressure conditions where dissolved inorganic carbon dynamics and metabolic regulation become increasingly important [118]. Furthermore, the long-term geomechanical stability and pore-network evolution under repeated Sc-CO_2_ exposure warrant further investigation through extended laboratory validation and pilot-scale assessment [117]. Addressing these constraints through improved injection control strategies, adaptive microbial management, and integrated reservoir modeling remains a key prerequisite for advancing practical implementation. Under appropriately managed reservoir and biogeochemical conditions, integrated workflows provide a conceptual and technical foundation for exploring scalable CBM production systems, with the potential to enhance methane recovery while contributing to reduced net carbon emissions through coupled geological CO_2_ sequestration and biological conversion processes.

## 6. Conclusions and Prospects

The core scientific objective of coalbed gas bioengineering is to enhance CBM reserves through regulated microbial metabolic processes, while concurrently improving key reservoir properties. This approach is conceptually based on the strategic introduction of functionally optimized methanogenic consortia as biological stimulants, coupled with CO_2_ co-injection to synergistically activate hydrogenotrophic methanogenic pathways and sustain microbial activity within coal reservoirs. Despite its conceptual and mechanistic advantages, the translation of this strategy into practical engineering applications remains constrained by a series of unresolved scientific and technical challenges.

### 6.1. Enhancing Coal Biodegradability and Evaluating the Potential for Engineering Applications

To enhance coal biodegradation efficiency, the selection of environmentally benign pretreatment strategies is critical for preserving subsurface microbial ecosystems. Although conventional acid–alkali pretreatment methods can improve coal reactivity, they often induce reservoir sensitivity damage and generate extreme pH conditions that severely compromise microbial viability. In contrast, recent advances in Sc-CO_2_ extraction have demonstrated a dual capacity to modify the physicochemical properties of coal while simultaneously increasing its bioavailability. This approach therefore represents a promising alternative for future application, provided that the remaining technical constraints can be effectively addressed. Moreover, deep CBM reservoirs offer strategic opportunities for coupling biomethane enhancement with geological carbon sequestration. Realizing this potential will depend on the rational selection of pilot sites and the systematic resolution of operational challenges through targeted engineering-scale trials.

### 6.2. Optimized Microbial Consortium Enrichment Engineering and Metabolic Network Regulation

The development of stage-specific microbial consortia through coordinated metabolic coupling during coal organic matter transformation represents a key frontier in subsurface biotechnology. Conventional enrichment strategies, which prioritize maximal metabolic performance under idealized laboratory conditions, often exhibit limited translational applicability when confronted with the physicochemical heterogeneity of subsurface coal reservoirs. Improving carbon utilization efficiency in extremophilic microbial consortia therefore requires targeted intervention at enzymatic bottlenecks, which in turn depends on accurate identification of rate-limiting biochemical pathways and functionally dominant microbial groups across distinct methanogenic stages. By integrating metabolomic and proteomic analyses to resolve variations in metabolic pathways and conversion efficiencies, high-performance microbial consortia adapted to in situ reservoir conditions can be selectively cultivated. In parallel, reservoir-specific on-site amplification of microbial communities, together with engineering-scale applicability testing, is essential to evaluate microbial acclimation behavior and optimize culture media formulations under representative subsurface conditions.

### 6.3. Integrated Scientific Framework and Engineering Paradigms for Microbial CO_2_ Sequestration

Under the imperative of carbon neutrality, the strategic integration of CBM bioengineering with Sc-CO_2_ extraction has emerged as a transformative pathway for subsurface carbon management. Carbon sequestration capacity and conversion efficiency constitute the core performance indicators of this approach, while their controlling factors and underlying mechanisms provide the scientific basis for engineering design and operational optimization. High-efficiency microbial consortia serve as the principal biological drivers of this process, and their metabolic performance can be enhanced through approaches including targeted strain enrichment, diversification of microbial sources, coordinated application of indigenous and exogenous microorganisms, and the incorporation of conductive materials to facilitate electron transfer. At present, research on in situ CO_2_ biosequestration in coal reservoirs remains largely confined to laboratory-scale mechanistic investigations and a limited number of pilot engineering tests. Advancing toward practical deployment requires large-scale field demonstrations that integrate site selection strategies, pipeline network configuration, biofracturing fluid formulation, CO_2_ injection schemes, and real-time monitoring and regulation of biogeochemical processes, thereby establishing a systematic and reliable CO_2_ biosequestration technology framework. Moreover, given the structural complexity and heterogeneity of deep coal reservoirs, comprehensive evaluation of the economic feasibility and environmental sustainability of CO_2_ biosequestration is essential. Such assessments should include analyses of carbon credit benefits alongside potential impacts on groundwater systems, ensuring that technological advancement aligns with long-term environmental stewardship.

## Data Availability

No new data were created or analyzed in this study. Data sharing is not applicable to this article.
